# Demographic, nutritional, social and environmental predictors of learning skills and depression in 20,000 Indian adolescents: Findings from the UDAYA survey

**DOI:** 10.1371/journal.pone.0240843

**Published:** 2020-10-16

**Authors:** Samuel Scott, Anjali Pant, Phuong Hong Nguyen, Sachin Shinde, Purnima Menon

**Affiliations:** 1 International Food Policy Research Institute, Washington, DC, United States of America; 2 International Food Policy Research Institute, New Delhi, India; 3 Population Council, New Delhi, India; National University of Singapore, SINGAPORE

## Abstract

**Objectives:**

Adolescent wellbeing is critical to breaking the intergenerational cycle of poverty and one in five of the world’s adolescents live in India. We explored predictors of learning skills and depression in Indian adolescents.

**Methods:**

Data on adolescents aged 10–19y (three groups: 5,840 unmarried males, 8,953 unmarried females, 4,933 married females) were available from the state-representative Understanding the Lives of Adolescents and Young Adults survey in Uttar Pradesh and Bihar. Multivariable logistic regression models adjusted for cluster sampling design and state fixed effects were used to examine factors (demographic, health/nutrition, social, and environmental) associated with three outcomes: reading proficiency, math proficiency, and depressive symptoms.

**Findings:**

Learning skills were poor (28–61% lacked basic reading and math skills depending on adolescent group and outcome) and depression was common (8–26%). Better learning skills were predicted by greater household wealth (AOR 1.72–2.55 depending on group) and household head education (AOR 1.03–1.07 per year), being in school (AOR 4.19–18.65), parental support (AOR 1.11–1.39), having gender equal attitudes (AOR 1.56–2.67), number of food groups consumed at least weekly (unmarried females: AOR 1.11), and having an improved latrine (AOR 1.33–1.51). Poorer learning skills were predicted by family substance use (AOR 0.68–0.74), underweight (males: AOR 0.74), witnessing parental violence (AOR 0.66–0.78). Depressive symptoms were predicted by witnessing parental violence (AOR 1.51–1.92) and experiencing sexual abuse (AOR 2.30–6.16).

**Conclusion:**

Factors across multiple life dimensions are associated with learning skills and depression in Indian adolescents. Adolescent-focused policies and programs should consider health/nutrition, social, and environmental aspects of life in vulnerable individuals.

## Introduction

Strategic investments in adolescent capabilities are highly cost effective and are critical for sustainable global development [1, 2]. Adolescents are one sixth of the world’s population—1.3 billion adolescents in 2019—and India is home to one fifth of the world’s adolescents [3].

Indian adolescents face a poor learning environment. While substantial progress has been made in school enrolment in India, the quality of education has worsened [4] and many adolescents do not complete secondary school [5]. The percentage of standard five students, approximately 11-year-olds, who could read a standard two-level text decreased from 53% in 2006 to 48% in 2014 [6]. Similarly, those who could solve division problems decreased from 43% in 2006 to 26% in 2014.

In addition to poor educational quality, adolescents in India face a high burden of common mental disorders including depression. In 2007, a survey found that 25% of adolescents felt sad/hopeless almost every day for two weeks or more in a row [7] and a 2015–2016 adolescent survey found a mental morbidity prevalence of 7% [8]. Though individual surveys recognize adolescent depression as a problem, better tools are needed to estimate the prevalence of adolescent mental health conditions at the population level for comparison to other low and middle income countries; UNICEF is currently leading an effort to develop such a tool [9]. As adolescents in India are soon to enter the workforce and are often parents themselves due to early childbearing, the consequences of poor mental health during adolescence are far-ranging and intergenerational [10, 11].

A two-fold relationship exists between educational and mental health outcomes in adolescents. Healthy young people learn better and have improved life chances [12], and more-educated young people have better mental health [13]. Building effective adolescent learning and health programs requires an evidence base on the determinants in the targeted context. Examining these determinants, however, is inherently difficult because learning and health are shaped by a complex set of factors at individual, family, social, and environmental levels. Surveys rarely collect information on most aspects of adolescents’ lives and often suffer from small sample size, narrow age inclusion, and omission of factors that may contribute to learning ability and mental health. The Understanding the Lives of Adolescents and Young Adults (UDAYA) survey provides a unique opportunity to fill these evidence gaps and inform adolescent-focused programs and policies [14, 15].

The objectives of the current paper were to 1) describe adolescent learning and mental health in northern India in terms of reading proficiency, math proficiency and depressive symptoms and 2) explore which factors—related to demographics, health/nutrition, social and family life, and living environment—predict these outcomes.

## Materials and methods

### Data source and study population

Data were from the Population Council’s UDAYA study in 2015–2016 [14, 15]. UDAYA collected data for 20,589 adolescents aged 10–19 years in Uttar Pradesh and Bihar states. These two states are large, highly populated, predominantly rural, high poverty states in northern India and account for 28% of the adolescent population in the country [14, 15]. We examined unmarried males aged 10–19 years, unmarried females aged 10–19 years, and married females aged 15–19 years. Our primary sample for analyses included 19,726 adolescents (after excluding 4.2% with missing data) and our secondary subsample included 7,627 adolescents with data on hemoglobin and body mass index (S1 Table). The primary data collection was approved by the Institutional Review Board of Population Council.

### Outcomes

We examined three outcomes: reading proficiency, math proficiency, and depression. Reading and math proficiency were assessed by the Annual Status of Education Report (ASER) tools [5], validated in India [16] and used in the ASER nationwide survey, repeated annually since 2005, to track progress in basic learning. The reading component assessed four levels of proficiency in the Hindi language: ability to recognize letters, read words, read a short paragraph (standard 1 level text; ages 6–7 years), and read a ‘story’ (standard 2 level text; ages 7–8 years). The math component assessed four levels of proficiency: ability to recognize single-digit numbers (1–9), double-digit numbers (11–99), solve two-digit subtraction problems (e.g., 46–29) and three-digit division problems (e.g., 879/7). Depressive symptoms were screened using the 9-item Patient Health Questionnaire (PHQ-9) [17]. The PHQ-9 has been validated in Indian adolescents and was found to be an effective tool to screen for depression in this setting and age group [18]. The PHQ-9 score ranges between 0 and 27, with higher scores indicating more severe symptoms of depression in the past two weeks. The outcomes in our study were dummy variables for ability to read a story, solve at least two subtraction problems, and a PHQ-9 score of 5 or more indicating any depression per the standard cutoff. All variable definitions are provided in S2 Table.

### Predictors

We chose predictors for the regression analysis primarily on the basis of the availability of data in the UDAYA surveys and the broader literature on risk factors for adolescent wellbeing [13]. Broadly, we examined risk factors that covered demographic, household, health and nutrition, social and environmental factors.

Demographic factors included age, religion, caste, household wealth, household head education, and state. A household wealth index was constructed using factor analysis of ownership of assets. The first factor derived from the analysis was used to classify adolescents into wealth quartiles.

Health and nutrition factors included anemia, thinness, dietary diversity, currently pregnant, and ever given birth. Hemoglobin (Hb) was measured in the field from a finger-prick blood sample using a portable Hemocue Hb 201+ instrument. Standard age-, sex-, and pregnancy-specific World Health Organization (WHO) cutoffs were applied to define anemia (S2 Table) [19]. Height was measured to the nearest 0.1 cm using a SECA 213 stadiometer and weight was measured to the nearest 100 grams using a SECA 874 electronic scale. Thinness for adolescents 10–18 years old was computed with the ‘zbmicat’ STATA function, which uses age- and gender-adjusted body mass index (BMI) cutoffs from the Childhood Obesity Working Group of the International Obesity Taskforce (IOTF) [20]. As IOTF cut-offs are applicable only to adolescents ≤18 years old, WHO BMI cut-offs were used for the 19-year-old adolescents [21], with thinness defined as having a BMI Z-score <-2 standard deviations from the reference. A food frequency questionnaire was used to capture dietary habits. We used the number of the following eight food groups consumed at least weekly: eggs, chicken/meat, fish, pulses/beans, dark green vegetables, other vegetables, fruits, and dairy. Pregnancy and birth indicators were only collected in married females.

Social factors included adolescents’ social networks, family relations, household social environment, experience of sexual abuse, and gender attitudes. Social networks were proxied by the number of friends and time spent with friends (often or not often). A parental support score of whether the adolescent discussed friendship, physical changes, leisure and personal matters with their parents ranged from 0–4. Other family/household indicators included whether the adolescent had similarly-aged family members (within three years of the adolescent’s age), substance (drugs, tobacco or alcohol) use by family members, and ever witnessing their father beating their mother. Sexual abuse was measured by whether the adolescent was ever deliberately touched on their private parts when they did not want to be touched. Gender attitudes were assessed by asking if girls should be allowed to decide when they want to marry, and if persons other than the husband/father should be able to decide how household money is to be spent. Our gender attitude indicator was a positive response for either item [22].

Environmental factors included residence (urban/rural), whether the household had an improved sanitation facility, school attendance (out of school, in government school, in private school), and whether the adolescent engaged in paid or unpaid work outside of school in the last year.

### Statistical analysis

Analyses were conducted separately for unmarried males, unmarried females, and married females to understand if associations varied between these three groups. Multivariable logistic regression models were used to examine associations between the predictors and each outcome for each group. The regression models included sample weights and were adjusted for clustered standard errors and state fixed effects to account for intra cluster correlation and state-specific unobservable factors. The models were repeated for the subsample with data on anemia and thinness; from these models, we only present results for anemia and thinness (not all predictors) given similar results for other predictors examined in the primary sample. Analyses were conducted in STATA software version 15.

## Results

### Sample characteristics

#### Demographic, health, social, and environmental factors

Adolescents were mostly Hindu, from backward castes (i.e. disadvantaged social groups) and poor households (Table 1). Anemia was higher but thinness was lower in females compared to males. Among married females, 22% were currently pregnant and 44% had given birth. Dietary diversity was suboptimal, with 4.4 of 8 food groups consumed at least weekly. Married females were far less likely to meet and spend time with their friends than unmarried adolescents. Adolescents typically discussed 2 of 4 aspects of their lives with their parents and most had a family member who used substances (mainly tobacco and alcohol). Witnessing parental violence and experiencing sexual abuse were reported more by females than males. Most adolescents expressed gender equal attitudes, though the percentage was slightly lower in males than females. Less than half of the adolescents were from households with improved latrine facilities. Compared to males, more females were out of school, fewer were in private schools, and fewer worked outside of school.

**Table 1:** Demographic, health, social and environmental characteristics of Indian adolescents aged 10–19 years.

	Male	Female	
	Unmarried (n = 5,840)	Unmarried (n = 8,953)	Married (n = 4,933)
Demographic			
Age, years	15.0 (2.63)	15.81 (2.22)	17.96 (1.09)
Aged 15–19 years	64.35	81.63	100
Hindu religion	82.28	72.75	83.86
Backward caste	79.93	77.17	89.88
Wealth index^1^	0.37 (0.26)	0.39 (0.26)	0.28 (0.21)
Household head education, years	5.78 (5.39)	5.47 (5.43)	3.86 (4.56)
Bihar	47.84	44.43	65.66
Health and nutrition			
Anemic^2^	27.39	58.52	66.89
Thinness^3^	45.99	38.93	30.74
Currently Pregnant	NA	0	21.55
Ever given birth	NA	0	44.37
Dietary diversity^4^	4.5 (1.54)	4.38 (1.66)	4.32 (1.70)
Social			
Number of friends	4.48 (3.84)	3.61 (2.88)	3.58 (2.78)
Often spends time with friends	86%	71%	9%
Parental support^5^	2.05 (1.03)	2.31 (0.90)	NA
Number similarly aged family^6^	1.00 (0.90)	1.15 (0.93)	0.96 (1.04)
Family substance use	70.53	70.18	76.44
Witnessed parental violence	17.43	23.63	29.76
Sexual abuse	1.56	8.16	9.22
Gender equal attitude^7^	81.83	91.11	90.78
Environmental			
Lives in urban setting	47.28	47.98	37.04
Improved latrine facility at home	42.72	46.02	25.93
Out of school	20.91	31.41	89.3
In government school	43.42	44.50	8.60
In private school	35.67	24.09	2.11
Work out of school in last year^8^	60.99	38.49	27.16

Values are means (SD) or percentages; for each row, groups with different letters are significantly different (p<0.05) using ANOVA test.

^1^Normalized score (0–1) using factor analysis of wealth index (electricity, fan, television, sewing machine, computer, fridge, clock, bicycle, motorbike, car, water pump, tractor, land ownership, cooking fuel type, house material).

^2^Anemia available for subsample (n = 3,140 unmarried males, 2,585 unmarried females, 1,902 married females); defined per WHO age- and sex-specific cutoffs (World Health Organization, 2011).

^3^Thinness available for subsample (n = 3,140 unmarried males, 2,585 unmarried females, 1,902 married females); defined using International Obesity Task Force cutoffs for those aged 10–18 years (Cole et al. 2012) and WHO criteria for those aged 19 years (World Health Organization, 2007).

^4^Number of food groups consumed at least once per week (out of 8 total).

^5^Normalized score (0–1) using factor analysis of multiple topics of discussion with parents (school performance, friendship, physical changes, leisure, personal matters).

^6^Similar aged family members defined as ±3 years.

^7^Dummy variable for ‘yes’ response to either 1) girls should be allowed to decide when they want to marry or 2) persons other than husband/father should be able to make household spending decisions.

^8^Either paid or unpaid work.

NA, not applicable.

#### Reading proficiency, math proficiency, depressive symptoms

**Fig 1:**
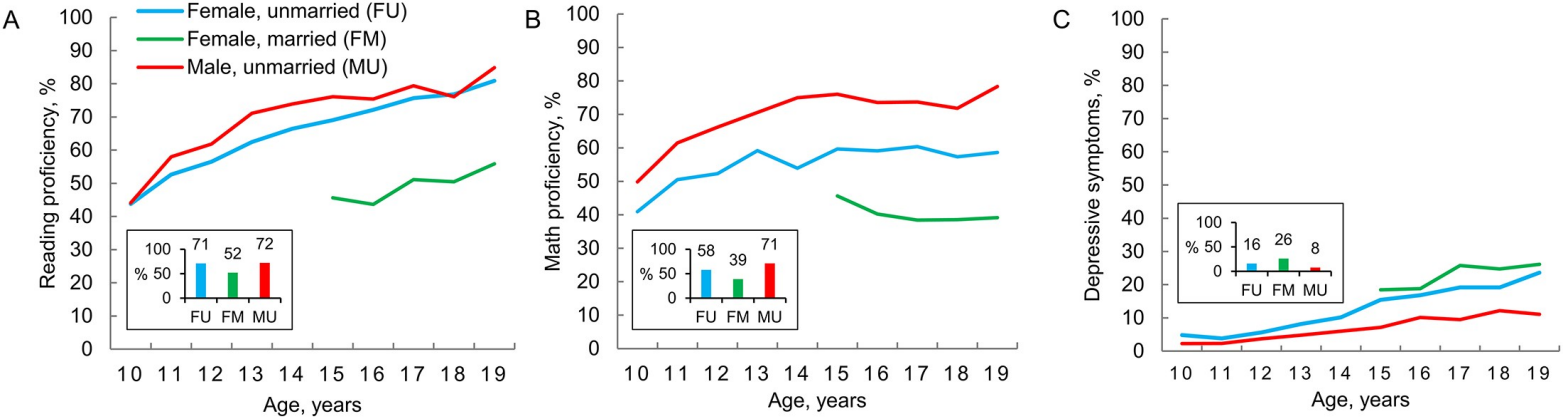
Reading proficiency, math proficiency, and depressive symptoms in Indian adolescents by age and sex. Reading (A) and math (B) proficiency represent the abilities to read a story and solve at least two subtraction problems per the Annual Status of Education Report tools. Depressive symptoms (C) are indicated by a score of at least 5 out of 27 on the Patient Health Questionnaire-9. Values in the charts are the mean percentages by year of age, with the group means for all ages combined shown in the insets.

Basic learning outcomes—reading and math proficiency—were poor. Overall, only 71% of unmarried females, 52% of married females and 72% of unmarried males were able to read a story (Fig 1A). Even by 19 years of age, 15–20% of unmarried adolescents and 44% of married female adolescents could not read a story (Fig 1A and S3 Table). Math proficiency was higher for males than unmarried or married females at all ages from 10 to 19 years (Fig 1B). Depressive symptoms were more common in older compares to younger adolescents and in females compared to males (Fig 1C). One in four married females reported at least mild depressive symptoms. Only 6% of females and 1% of males had moderate to severe depression, thus our outcome predominantly represents mild cases. The mean (SD) PHQ-9 scores were 1.1 (2.2) for unmarried males, 2.0 (3.6) for unmarried females, and 2.9 (4.3) for married females.

### Factors associated with learning ability and mental heath

**Fig 2:**
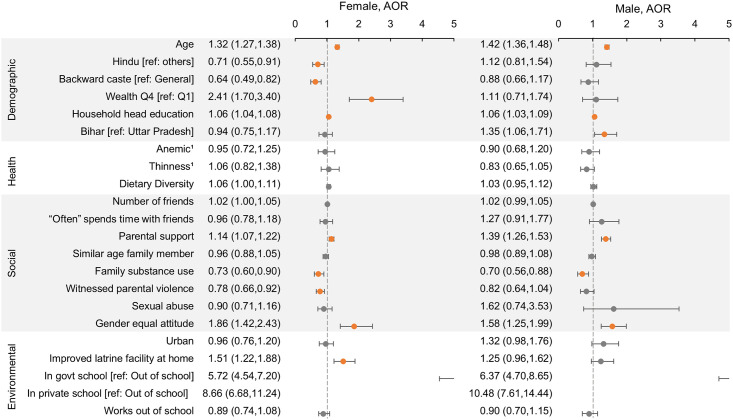
Factors associated with reading proficiency in unmarried Indian adolescents. Predictors of ability to read at story level using multivariable logistic regression models with cluster controls. Separate models were run for females and males. Adjusted odds ratios (AOR) and 95% confidence intervals (CI) are shown, with a reference line at 1.0. Orange point estimates are significant (CI does not include 1). The x-axis scale was restricted to a maximum of 5 for visual clarity; values above 5 are cut off. 1Data on anemia and thinness were only available for a subsample (see Methods); point estimates shown are from subsample analysis adjusted for all other factors.

**Fig 3:**
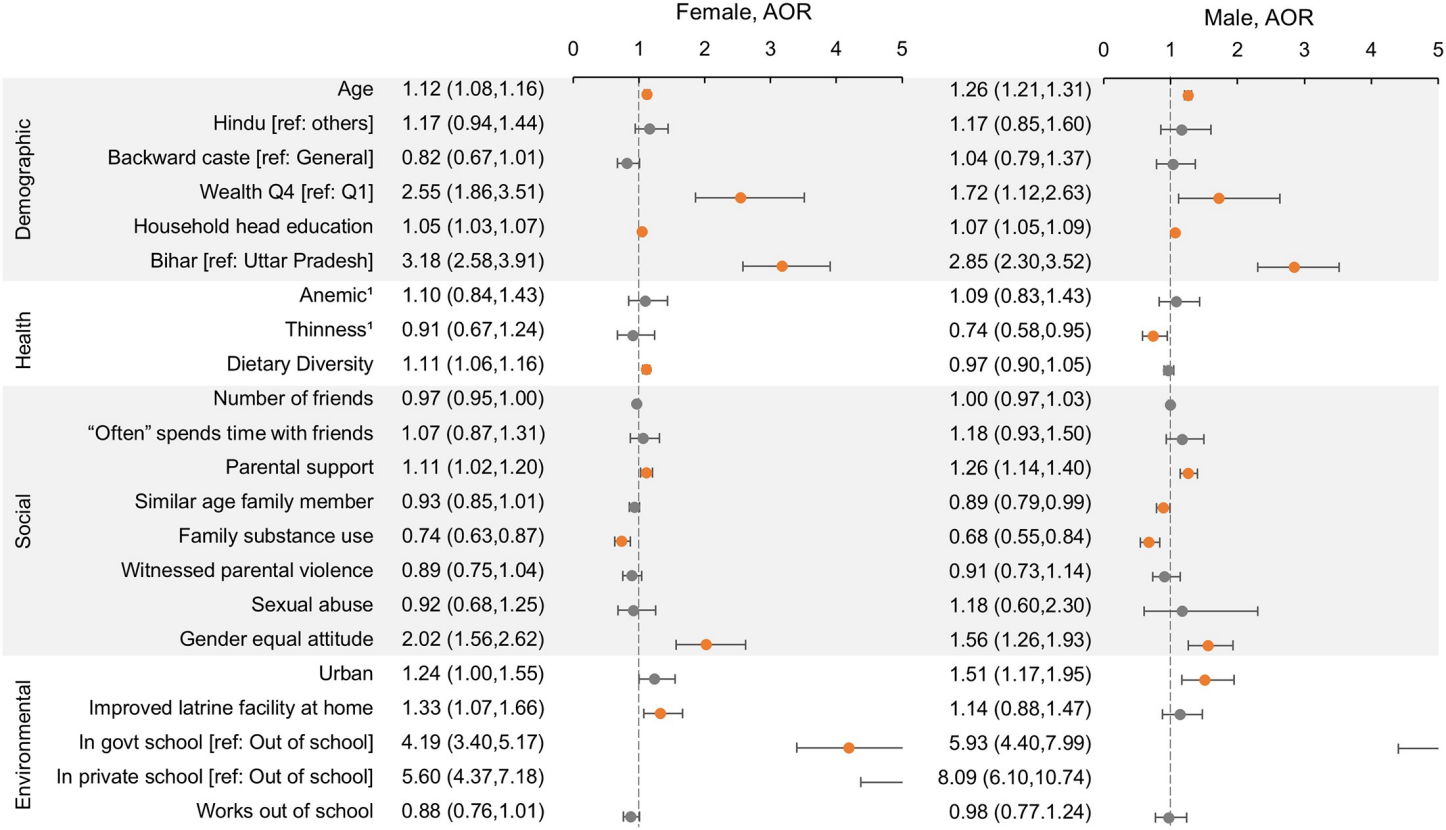
Factors associated with math proficiency in unmarried Indian adolescents. Predictors of ability to solve at least two subtraction problems using multivariable logistic regression models with cluster controls. Separate models were run for females and males. Adjusted odds ratios (AOR) and 95% confidence intervals (CI) are shown, with a reference line at 1.0. Orange point estimates are significant (CI does not include 1). The x-axis scale was restricted to a maximum of 5 for visual clarity; values above 5 are cut off. 1Data on anemia and thinness were only available for a subsample (see Methods); point estimates shown are from subsample analysis adjusted for all other factors.

**Fig 4:**
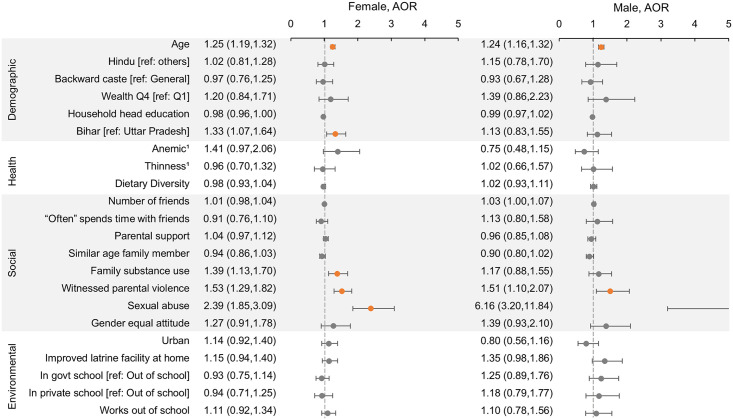
Factors associated with depression in unmarried Indian adolescents. Predictors of depressive symptoms using multivariable logistic regression models with cluster controls. Separate models were run for females and males. Adjusted odds ratios (AOR) and 95% confidence intervals (CI) are shown, with a reference line at 1.0. Orange point estimates are significant (CI does not include 1). The x-axis scale was restricted to a maximum of 5 for visual clarity; values above 5 are cut off. 1Data on anemia and thinness were only available for a subsample (see Methods); point estimates shown are from subsample analysis adjusted for all other factors.

Increasing age, being from a relatively wealthy household, and higher education of the household head were associated with higher reading and math proficiency (Figs 2 and 3; adjusted odds ratios and 95% confidence intervals shown in figures). Increasing age was also predictive of depression (Fig 4; S4 Table). In unmarried females, Hindu religion and backward caste predicted poorer reading proficiency.

Thinness was associated with poorer math proficiency among males and consuming more food groups was associated with better math proficiency among unmarried females (Fig 3). In married females, being pregnant was associated with increased odds of depression (S4 Table). Anemia was unrelated to the outcomes after adjustment for other factors.

Friendships—in terms of number of friends or time spent with friends—were generally not associated with the studied outcomes. A higher parental support score and having a gender equal attitude, however, were both strongly predictive of better reading and math proficiency (Figs 2 and 3). In contrast, use of drugs, tobacco, or alcohol by family members and witnessing parental violence were both predictive of poorer reading and math scores. Family substance use, witnessing parental violence, and experiencing sexual abuse were all associated with higher odds of depression (Fig 4).

Living in an urban environment was associated with higher math proficiency among adolescents but showed no association with other outcomes. Having an improved latrine facility at home was associated with higher reading and math in unmarried females but not in males. The strongest predictor of reading and math proficiency among all factors considered was being in school, with those in private school having higher scores than those in government school. Engaging in labor outside of school in the previous year was predictive of poorer reading in married females only (S4 Table). Environmental factors were not predictive of depressive symptoms.

## Discussion

We examined factors across multiple dimensions associated with basic learning outcomes and depressive symptoms among Indian adolescents. We found that better performance on math and reading tests was predicted by being from an advantaged caste, from a relatively wealthy home with a more educated household head and an improved latrine, in school (especially private school), having open communication with parents, and having gender equal attitudes. Depressive symptoms were higher among those who had witnessed parental violence and experienced sexual abuse.

Being from a backward caste predicted lower reading ability in females but not in males. Son preference is a well-described issue in India, especially among socially disadvantaged groups [23]; males in backward castes may be given preference to attend school, and thus the caste-reading association only exists for females. Though demographic factors are non-modifiable and generally treated as confounders, understanding how they relate to outcomes is important for future program targeting. Our findings suggest that low-caste females from poor households with poorly-educated parents should be a target group for programs to improve reading proficiency. In contrast, demographic factors were largely unrelated to depression, suggesting that mental wellbeing issues cut across the spectrum of social privilege.

The generally weak associations do not imply that health and nutrition is unimportant in terms of academic performance or mental health but rather that, when taking a holistic view of adolescents’ lives in this high poverty context, other factors appear to be more important. A limitation in our case were relatively crude measures of health and nutrition available in the data. Previous work has shown associations between academic or cognitive outcomes and more specific measures such as diet quality measured through a food frequency questionnaire or 24-hour recall [24], biomarkers of micronutrient status or aerobic fitness [25].

We found that an interactive relationship between the adolescent and her/his parents was predictive of reading and math proficiency but not depression. Often spending time with friends was predictive of lower likelihood of depression in males, possibly a coping strategy. Other work has highlighted the importance of social support for adolescent wellbeing. In an analysis of Global School-Based Health Surveys from India, Sri Lanka, Pakistan, and Myanmar, depressive symptoms were less likely in those with close friendships and involved parents [26].

Gender-equal views were positively associated with learning outcomes. In Bihar, learning outcomes were better in adolescents whose teachers had gender equal attitudes [27], thus a learning environment where teachers and students believe in equal treatment of boys and girls is related to better learning outcomes. However, we found that gender equal attitudes predicted a two-fold higher risk of depression in married females. After marriage, women in rural India typically move into the home of their spouse’s family where, if they retaliate against traditional gender roles, they could face social challenges. This finding could reflect a frustration with traditional gender roles among young women with more egalitarian views.

Substance abuse by family members, ever witnessing parental violence, and ever experiencing sexual abuse were related to poorer outcomes, especially depression. In Maharashtra, harsh parental behavior, disturbed family relationships, and substance abuse were predictive of poor adolescent mental health [28]. A systematic review and meta-analysis from 21 countries found that girls who experienced sexual violence had a three-fold increased risk of school absenteeism and children who witnessed parental violence scored lower on standardized tests [29]. These findings are also in line with a recent study of adolescents in northern India which found that childhood maltreatment was related to poor mental health in adolescence [30].

The strongest predictor of math and reading ability was attending school, with an added advantage of private over government school. The UDAYA survey is unique among most adolescent surveys in that it sampled households rather than schools, thus capturing out-of-school adolescents. Older adolescents drop out of school in this context for various reasons including early marriage, to help with household chores or to work, inability to pay tuition fees, a lack of secondary schools, or living too far away from the school [31]. Having an improved latrine facility predicted higher reading and math ability in females but not in males. An improved latrine could benefit females through greater privacy, lower risk of uro-genital tract infections, and protection from assaults by men [32]. Soil-transmitted helminth infection has been associated with cognitive and educational deficits in school-aged children [33].

As primary school enrolment is now nearly universal in India, improving educational quality, providing adolescents with supportive home and social environments, and promoting secondary school completion are important next steps in achieving better learning outcomes. ASER surveys show declining school performance in India over time [4], which could be for several reasons. As enrolment increases, teacher resources are strained. Additionally, the last to enroll are the most marginalized groups whose performance is likely the worst. Investments into teacher training and school infrastructure have not been adequate to accommodate the increase in enrolment in the last two decades, with most financial resources in the country being used for enhancing higher education (universities) rather than basic K-12 education [34]. The convergence of educational and health policies is critical for achieving adolescent wellbeing [13]. Expansion of secondary education provides a range of indirect health benefits for young people as a social determinant of health and as a platform to deliver health goods and interventions. In many countries, health-education interfaces such as nutrition programs through schools are withdrawn just as children reach puberty, a growth period with increased nutritional needs. Policy interventions that factor in social norms and target multiple drivers of change have been effective in improving educational quality and student performance in developing countries [35]. In India, examples include out-of-school literacy programs [36], teacher feedback [37], teacher pay incentives [38], and remedial and computer-assisted learning [39].

Our study is not without limitations. The multivariable models adjusted for 24 different factors across multiple dimensions of life in a state-representative sample of male and female adolescents of all ages, in and out of school, unmarried and married. Though this approach reduces potential confounding, with cross-sectional data we are not able to identify causal associations or account for previous life experiences that may have affected the studied outcomes. Second, as mentioned, dietary and nutritional data were limited. Third, we were unable to examine predictors of learning outcomes and mental health in married males given that this group was not included in the UDAYA survey.

In conclusion, our findings highlight the importance of addressing the social determinants of mental health in adolescents [40]. While a program designed to keep adolescents in school may benefit academic performance, it may not be sufficient to prevent or address depression. From a curative perspective, a major barrier that needs to be overcome in India is inadequate access to mental health resources. The 2015–16 National Mental Health Survey of India revealed a treatment gap of 85% for common mental disorders among adults [8]. For adolescents, a similar situation of poor access to services is illustrated by a survey of Bangladeshi adolescents; among those with depression, 80% sought no help [41]. India will not be able to meet its development goals without building the education, skills, productivity, and health of its youth population. Our findings imply that building adolescent capabilities and mental health in this context requires a holistic approach targeting health, social, and environmental factors in the most vulnerable individuals.

## Supporting information

S1 Table Sample selection steps. (DOCX)

S2 Table Variable definitions. (DOCX)

S3 Table Prevalence of reading proficiency, math proficiency, and depressive symptoms by age for unmarried female, married female, and unmarried male Indian adolescents. (DOCX)

S4 Table Factors associated with reading proficiency, math proficiency, and depressive symptoms in adolescent Indians—married females 15–19 years. (DOCX)

S1 Dataset (DTA)

S2 Dataset (DTA)
