# Supplementary material for: Demographic, nutritional, social and environmental predictors of learning skills and depression in 20,000 Indian adolescents: Findings from the UDAYA survey
Source: PLoS One. 2020 Oct 16;15(10):e0240843. doi: 10.1371/journal.pone.0240843 (PMC7567371; doi:10.1371/journal.pone.0240843)
Supplement: S1 Table — (DOCX) [file pone.0240843.s001.docx]

| **S1 Table. Sample selection steps** | | | |
| --- | --- | --- | --- |
|  | **Males - unmarried** | **Females - unmarried** | **Females - married** |
| Overall UDAYA sample (not analysed) |  |  |  |
| Reading | 5764 | 8795 | 3783 |
| Math | 5764 | 8795 | 3783 |
| Depression | 5967 | 9418 | 5204 |
| 🡪Excluded due to missing data on outcomes or predictors (except health predictors) | | | |
| 🡪Reading | 🡪127 | 🡪460 | 🡪270 |
| 🡪Math | 🡪127 | 🡪460 | 🡪270 |
| 🡪Depression | 🡪127 | 🡪465 | 🡪271 |
| Primary sample for analysis |  |  |  |
| Reading | 5637 | 8335 | 3513 |
| Math | 5637 | 8335 | 3513 |
| Depression | 5840 | 8953 | 4933 |
| 🡪Excluded due to no health data (Missing data on hemoglobin and body mass index) | | | |
| 🡪Reading | 🡪2583 | 🡪5883 | 🡪2152 |
| 🡪Math | 🡪2583 | 🡪5883 | 🡪2152 |
| 🡪Depression | 🡪2700 | 🡪6368 | 🡪3031 |
| Secondary health subsample for analysis |  |  |  |
| Reading | 3054 | 2452 | 1361 |
| Math | 3054 | 2452 | 1361 |
| Depression | 3140 | 2585 | 1902 |
