# Supplementary material for: Demographic, nutritional, social and environmental predictors of learning skills and depression in 20,000 Indian adolescents: Findings from the UDAYA survey
Source: PLoS One. 2020 Oct 16;15(10):e0240843. doi: 10.1371/journal.pone.0240843 (PMC7567371; doi:10.1371/journal.pone.0240843)
Supplement: S2 Table — (DOCX) [file pone.0240843.s002.docx]

| S2 Table. Variable definitions | |
| --- | --- |
| Indicator | Definition |
| Outcomes |  |
| Literacy | Respondent able to read at story level (Hindi language) |
| Numeracy | Respondent able to solve at least two problems of subtraction. |
| Depression | Respondent suffering from any form of depression- mild, moderate, moderately severe or severe. PHQ-9 score values are used to identify depression levels among respondents. [Criteria used are as follows](https://www.ncbi.nlm.nih.gov/pmc/articles/PMC1495268/):   - Mild Depression: Score 5-9 - Moderate Depression: Score 10-14 - Moderately Severe Depression: Score 15-19 - Severe Depression: Score 20-27 |
| Demographic factors |  |
| Age 15-19 y [ref: 10-14y] | Age of respondent dummy, where base category refers to respondents in age bracket 10-14 years, and main category refers to respondents in age bracket 15-19 years. |
| Married | Respondent is married (both with and without gauna). |
| Hindu [ref: others] | Respondent belongs to Hindu religion. Here, reference category refers to respondents who belong to other religions including Muslim, Christian, Sikh, Buddhist and Jain. |
| Backward caste [ref: general caste] | Respondent belongs to a backward caste which includes scheduled caste (SC), scheduled tribe (ST) and other backward class (OBC). Here, reference category refers to respondents who belong to a general caste. |
| Household head education | Highest level of schooling completed by the head of the household (in years). |
| Bihar [ref: UP] | State Dummy, where base category is Uttar Pradesh and main category is Bihar. |
| Health factors |  |
| Anemic | The dummy of any anemia presence (mild, moderate or severe) is constructed using [WHO guidelines](https://www.who.int/vmnis/indicators/haemoglobin.pdf).   - Children aged 11 years or younger are considered anaemic if their Hb level is less than 115 g/l. - Children in age bracket 12-14 years and non-pregnant women (>=15 years) are considered anaemic if their Hb level is less than 120 g/l. - Pregnant women (>=15 years) are considered anaemic if their Hb level is less than 110 g/l.   Men (>=15 years) are considered anaemic if their Hb level is less than 130 g/l. |
| Underweight | Respondent is underweight (dummy). Here, base category refers to respondents either with normal or overweight (or obese). BMI (kg/m^2^) is calculated as:  BMI=weight/(height^2/10000)   - This BMI value is used to classify adolescents into the categories of underweight, normal weight and overweight. For adolescents in the age bracket 10-18 years[, zbmicat() of zanthro package](http://www.biostat.jhsph.edu/~courses/bio624/misc/STATA%20article%20on%20anthropometry%20measures.pdf) in Stata 15 is used for underweight classification based on Childhood Obesity Working Group of the International Obesity Taskforce cut-offs. For adolescents aged 19 years, [WHO criteria](http://www.euro.who.int/en/health-topics/disease-prevention/nutrition/a-healthy-lifestyle/body-mass-index-bmi) is used. |
| Currently Pregnant | Female respondent is currently pregnant. |
| Atleast one live birth | Female respondent ever given birth to atleast one live child. |
| Dietary Diversity | Total number of food groups consumed at least once a week. The list of food groups constitutes eggs, chicken or meat, fish, pulses or beans, dark vegetables, fruits, dairy (milk/curd) and vegetables other than greens. Total 8 food groups. |
| Social factors |  |
| Friends | Respondent’s total number of friends |
| Often spend time with friends | Dummy of whether respondent often spends time with his/her friends. Here, reference category constitutes respondents who either spend less time or no time with their friends. |
| Parental Support | Summative score of discussion with parents regarding multiple topics such as friendship, physical changes, leisure and other personal matters. This composite score ranges between 0 and 4. |
| Number of similarly aged family members | Respondent has a similar age (+ 3y) family member in the household. The indicator is constructed using household roster information of family members. |
| Family member takes  drugs/tobacco/alcohol | Respondent’s family member consumes either tobacco, alcohol or drugs. |
| Witnessed physical violence in house | Respondent’s father ever beaten his/her mother. |
| Sexual abuse | Respondent touched (when didn’t want to be touch) in a bad way. For example, touched on a private body part. |
| Spoken against gender disadvantage | Respondent showed support to atleast one of the gender disadvantage related issues. The issues are as follows:  Marriage: Girls should be allowed to decide when they want to marry.  Male dominance: Only husband or father alone should not decide how household money is to be spent. |
| Environmental factors |  |
| Urban [ref: rural] | Urban residence. Here, reference category is rural residence. |
| Wealth quartile | Respondents classified into four categories based on their wealth index. This index is a normalized score computed using factor analysis of wealth indicators including electricity, electric fan, tv, sewing machine, computer, fridge, clock, bicycle, bike, car, pump, tractor, land ownership, clean fuel, pucca and semi-pucca house. |
| Improved latrine facility | Availability of own flush toilet or own pit toilet. The indicator is constructed based on [UNICEF definition](https://www.unicef.org/progressforchildren/2006n5/index_35533.htm) of an improved latrine facility. |
| School Type | A dummy of school type is created with three levels- currently in a government school (level one), currently in a private school (level two), and out of school, that is currently out of school+never attended school (base level). |
| Paid or unpaid work  done in last 1 year | Respondent worked for any paid or unpaid job in the last one year. |
