# Supplementary material for: Demographic, nutritional, social and environmental predictors of learning skills and depression in 20,000 Indian adolescents: Findings from the UDAYA survey
Source: PLoS One. 2020 Oct 16;15(10):e0240843. doi: 10.1371/journal.pone.0240843 (PMC7567371; doi:10.1371/journal.pone.0240843)
Supplement: S3 Table — (DOCX) [file pone.0240843.s003.docx]

| **S3 Table. Prevalence of reading proficiency, math proficiency, and depressive symptoms by age for unmarried female, married female, and unmarried male Indian adolescents** | | | | | | | | | |
| --- | --- | --- | --- | --- | --- | --- | --- | --- | --- |
|  | **Reading proficiency, %** | | | **Math proficiency, %** | | | **Depressive symptoms, %** | | |
| Age, years | Female, unmarried | Female, married | Male, unmarried | Female, unmarried | Female, married | Male, unmarried | Female, unmarried | Female, married | Male, unmarried |
| 10 | 44 | NA | 44 | 41 | NA | 50 | 5 | NA | 2 |
| 11 | 53 | NA | 58 | 51 | NA | 62 | 4 | NA | 2 |
| 12 | 57 | NA | 62 | 52 | NA | 66 | 6 | NA | 4 |
| 13 | 62 | NA | 71 | 59 | NA | 71 | 8 | NA | 5 |
| 14 | 66 | NA | 74 | 54 | NA | 75 | 10 | NA | 6 |
| 15 | 69 | 46 | 76 | 60 | 46 | 76 | 15 | 18 | 7 |
| 16 | 72 | 44 | 75 | 59 | 40 | 74 | 17 | 19 | 10 |
| 17 | 76 | 51 | 79 | 60 | 38 | 74 | 19 | 26 | 9 |
| 18 | 77 | 50 | 76 | 57 | 39 | 72 | 19 | 25 | 12 |
| 19 | 81 | 56 | 85 | 59 | 39 | 78 | 24 | 26 | 11 |
| Reading and math proficiency represent the abilities to read a story and solve at least two subtraction problems per the Annual Status of Education Report tools. Depressive symptoms are indicated by a score of at least 5 out of 27 on the Patient Health Questionnaire-9. NA, not applicable because data were not collected for married females aged 10-14 years. | | | | | | | | | |
