# Supplementary material for: Demographic, nutritional, social and environmental predictors of learning skills and depression in 20,000 Indian adolescents: Findings from the UDAYA survey
Source: PLoS One. 2020 Oct 16;15(10):e0240843. doi: 10.1371/journal.pone.0240843 (PMC7567371; doi:10.1371/journal.pone.0240843)
Supplement: S4 Table — (DOCX) [file pone.0240843.s004.docx]

| **S4 Table. Factors associated with reading proficiency, math proficiency, and depressive symptoms in adolescent Indians – married females 15-19 years** | | | | | | |
| --- | --- | --- | --- | --- | --- | --- |
|  | Reading proficiency | | Math proficiency | | Depressive symptoms | |
|  | *AOR* | *95%CI* | *AOR* | *95%CI* | *AOR* | *95%CI* |
| Demographic |  |  |  |  |  |  |
| Age | 1.21** | 1.08,1.36 | 1.14* | 1.00,1.31 | 1.12* | 1.00,1.25 |
| Hindu [ref: others] | 0.75+ | 0.54,1.05 | 1.29 | 0.83,1.99 | 0.73* | 0.55,0.98 |
| Backward caste [ref: General] | 0.81 | 0.54,1.21 | 0.84 | 0.55,1.29 | 0.87 | 0.61,1.24 |
| Household head education | 1.04** | 1.01,1.06 | 1.03* | 1.01,1.06 | 1.00 | 0.97,1.02 |
| Wealth Q4 [ref: Q1] | 2.06** | 1.30,3.27 | 2.20** | 1.30,3.74 | 1.35 | 0.90,2.01 |
| Bihar [ref: Uttar Pradesh] | 0.93 | 0.72,1.20 | 3.64*** | 2.76,4.80 | 0.82 | 0.62,1.08 |
| Health |  |  |  |  |  |  |
| Currently pregnant | 0.81 | 0.63,1.05 | 0.81 | 0.62,1.05 | 1.61*** | 1.28,2.03 |
| Ever given birth | 1.13 | 0.92,1.40 | 0.91 | 0.70,1.17 | 1.02 | 0.82,1.26 |
| Dietary diversity | 1.08+ | 0.99,1.17 | 1.05 | 0.99,1.12 | 0.96 | 0.90,1.03 |
| Social |  |  |  |  |  |  |
| Number of friends | 1.03 | 0.99,1.07 | 0.99 | 0.95,1.03 | 1.06** | 1.02,1.09 |
| “Often” spends time with friends | 0.98 | 0.67,1.42 | 0.92 | 0.60,1.42 | 0.94 | 0.64,1.36 |
| Parental support |  |  |  |  |  |  |
| Number similarly aged family | 1.05 | 0.95,1.17 | 1.06 | 0.95,1.18 | 0.97 | 0.88,1.06 |
| Family substance use | 0.95 | 0.76,1.18 | 0.92 | 0.71,1.20 | 1.20 | 0.92,1.56 |
| Father has ever beaten mother | 0.80+ | 0.62,1.03 | 0.66** | 0.49,0.87 | 1.92*** | 1.52,2.43 |
| Sexual abuse | 1.36 | 0.93,2.00 | 1.28 | 0.77,2.14 | 2.30*** | 1.65,3.19 |
| Gender equal attitude | 2.67*** | 1.75,4.07 | 1.46 | 0.76,2.79 | 2.16*** | 1.49,3.13 |
| Environmental |  |  |  |  |  |  |
| Urban | 0.87 | 0.65,1.18 | 1.08 | 0.83,1.40 | 0.85 | 0.63,1.14 |
| Improved latrine | 1.13 | 0.84,1.52 | 1.17 | 0.81,1.68 | 0.96 | 0.73,1.27 |
| In government school [ref: Out of school] | 7.95*** | 5.15,12.26 | 6.50*** | 4.16,10.17 | 1.06 | 0.73,1.53 |
| In private school [ref: Out of school] | 18.65*** | 5.83,59.66 | 5.27*** | 2.99,9.29 | 0.55 | 0.23,1.33 |
| Work out of school in last year | 0.64*** | 0.50,0.83 | 0.86 | 0.67,1.09 | 1.07 | 0.84,1.35 |
| pseudo *R*^2^ | 0.150 |  | 0.213 |  | 0.085 |  |
| p | 0.00 |  | 0.00 |  | 0.00 |  |
| *N* | 3513 |  | 3513 |  | 4933 |  |
| +p<0.1, *p<0.05, **p<0.01, ***p<0.001  Adjusted odds ratios and 95% confidence intervals from multivariable logistic regression model with cluster controls. An individual model was run for each of the three outcomes. | | | | | | |
